# Working as a Healthcare Professional and Wellbeing During the COVID-19 Pandemic: Work Recovery Experiences and Need for Recovery as Mediators

**DOI:** 10.3389/fpsyg.2022.718422

**Published:** 2022-03-14

**Authors:** Claudia Lenuţa Rus, Cătălina Oţoiu, Adriana Smaranda Băban, Cristina Vâjâean, Angelos P. Kassianos, Maria Karekla, Andrew T. Gloster

**Affiliations:** ^1^Work and Organizational Psychology Research Center, Department of Psychology, Babeş-Bolyai University, Cluj-Napoca, Romania; ^2^Department of Psychology, Babeş-Bolyai University, Cluj-Napoca, Romania; ^3^Department of Applied Health Research, University College London, London, United Kingdom; ^4^Department of Psychology, University of Cyprus, Nicosia, Cyprus; ^5^Division of Clinical Psychology and Intervention Science, Department of Psychology, University of Basel, Basel, Switzerland

**Keywords:** healthcare professionals, recovery from work experiences, need for recovery, psychological wellbeing, serial mediation, emotional wellbeing

## Abstract

Considering the high impact strain that the severe acute respiratory syndrome coronavirus 2 (SARS-CoV-2) pandemic has put on medical personnel worldwide, identifying means to alleviate stress on healthcare professionals and to boost their subjective and psychological wellbeing is more relevant than ever. This study investigates the extent to which the relationships between the status of working in healthcare and the subjective and psychological wellbeing are serially mediated by work recovery experiences and the need for recovery. Data were collected from 217 Romanian employees (44 health professionals and 173 employees from other domains) using a cross-sectional design with self-report instruments, during the first stage of the nationwide lockdown. The results of the serial mediation analyses revealed that working in the medical field is indirectly related to subjective and psychological wellbeing through the following: (i) mastery experiences and (ii) mastery experiences as an antecedent of the need for recovery. As such, our findings indicate that (i) working in the medical field is, in fact, linked to healthcare professionals’ subjective and psychological wellbeing, and they provide some input on (ii) how recovery experiences and the need for recovery intervene in these relationships. Based on these findings, theoretical, methodological, and practical implications were suggested, and future research directions were proposed to maximize healthcare professionals’ wellbeing.

## Introduction

Literature shows that, in normal circumstances, healthcare professionals report longer working hours, less time spent in leisure activities, and shorter amounts of sleep, as compared to an average adult in other working environments (Cranley et al., 2016). What has been happening in hospitals all around the world for the past 2 years, due to the coronavirus disease 2019 (COVID-19) pandemic, has been far from “normal circumstances.” Medical staff is generally used to working under high-intensity conditions, and, therefore, they may have a higher psychological capacity to deal with the job strains associated with an epidemic (Teng et al., 2020) as compared to other professionals. However, the COVID-19 pandemic has increased the pressure on medical staff enough that such individual psychological resources have been stretched to the extreme. Stress levels, fatigue, burnout, and mental health problems among medical personnel have been shown to increase during the pandemic (Sagherian et al., 2020; Sasangohar et al., 2020), especially severe in its initial stages (Lai et al., 2020; Xu et al., 2020). This was true both for frontline medical staff (those working directly with patients with COVID-19) and for those working on their regular wards for uninfected patients (Wu et al., 2020). The extra strain of fear for loved ones, or being away from them, has added to the emotional pressures of their daily lives. In this context, there was less time for basic rest, i.e., let alone for the opportunity to engage in other activities they used to find comfort in, before the pandemic.

In their effort-recuperation model, Meijman and Mulder (1998) suggested a dynamic relationship between work-related effort and the potential positive or negative emotional, cognitive, and behavioral effects it has on an individual’s health and wellbeing. These symptoms of increased effort can be reversed or recuperated either during the same workday or the following night through a mechanism of replenishing one’s resources. This process is called work recovery, and it implies changes in physiological and psychological strain levels, as a result of leisure activities and non-work experiences that reduce strain and replenish resources (Sonnentag and Fritz, 2018). The key then to maintaining employee wellbeing is to ensure that resources are being promptly replenished at the end of a workday.

There has been consistent research interest in recovery mechanisms and ways to unwind from work demands in the last decade (Sonnentag and Fritz, 2018). However, recent studies emphasize the need to look into distinct work recovery-related constructs, in general, and work recovery experiences, in particular, within the specific context of healthcare professionals (Rus et al., 2020; Sonnentag et al., 2022). In their study, Rus et al. (2020) integrated the existing literature on work recovery in healthcare settings and offered an argument on how involvement in work recovery experiences could play an important role in maintaining healthcare professionals’ wellbeing and buffering the negative effects of increased job strain and continued effort.

Work recovery experiences are not equivalent to a specific activity *per se* but rather to the psychological experience of that activity, which leaves the individual feeling rejuvenated and energetic (Sonnentag and Fritz, 2007). Among the many potential such recovery experiences, four such experiences play an important role, namely, psychological detachment, relaxation, mastery experiences, and control (Sonnentag and Fritz, 2007). Psychological detachment implies refraining from work-related activities, thoughts, and emotions, once the workday is done (Sonnentag and Fritz, 2018). Relaxation involves a state of low mental and physical exertion (Sonnentag and Fritz, 2018). Mastery refers to involvement in off-job activities that provide challenging experiences and learning opportunities in other fields than one’s work domain (Sonnentag and Geurts, 2009). Control describes the degree to which a person can decide which activity to pursue during leisure time, as well as when and how to pursue this activity (Sonnentag and Fritz, 2007).

According to the broaden-and-build theory (Fredrickson, 1998, 2001), positive emotions play an important role in increasing the frequency with which individuals take part in recovery experiences after work. Positive emotions can temporarily broaden an individual’s perspective; they create affordances for accessing a larger pool of ideas and activities, which in turn generate and build upon the individual’s emotional and cognitive resources. This process creates an upward positive spiral, which would suggest that positive emotions are maintained through these recovery experiences and would continue to afford new opportunities for recovery of personal resources (Fredrickson and Joiner, 2018). Consequently, employees would more likely take on a proactive attitude toward uncovering ways to replenish their resources both at work and outside of it. However, in the absence of such a positive spiral, when the work effort increases to an extent where individuals do not take the time or have the energy to look for and take part in recovery experiences, we expect a constant level of perceived strain, lack of energy, and feelings of overload. The extent to which work generates a need to recuperate has been conceptualized as the *need for recovery* (van Veldhoven and Broersen, 2003). Hence, the need for recovery is indicative of short-term work fatigue, and it is considered an aspect of impaired wellbeing (Sonnentag and Fritz, 2007).

Wellbeing is not a one-dimensional construct. In his theory on positive mental health, Keyes (2002) distinguishes three wellbeing dimensions. Emotional wellbeing includes satisfaction and happiness with life and positive affect. Psychological wellbeing refers to the extent to which people thrive in their personal lives. Similarly, social wellbeing is a measure of an individual’s satisfaction with their social life (Robitschek and Keyes, 2009).

In the last decade, consistent research was dedicated to the need for recovery and recovery mechanisms and ways to unwind from work demands in relation to wellbeing (Sonnentag and Fritz, 2018; Wentz et al., 2020; Steed et al., 2021). These studies focus mainly on emotional or subjective wellbeing (Sonnentag et al., 2022). At present, there are no studies that integrate distinct work recovery-related concepts and multiple dimensions of wellbeing as an indicator of positive mental health, although the literature includes such a call for research (Steed et al., 2021). In line with the trend in recent literature to call for a systemic approach to the research of wellbeing and burnout (Montgomery et al., 2019), we argue that future work should look into multiple dimensions of wellbeing (Keyes, 2002) and the ways in which they are affected by both needs for recovery and recovery experiences.

The COVID-19 pandemic has emphasized the role of healthcare professionals in facing and overcoming a worldwide crisis. With higher levels of strain and solicited effort, there is an urgent need to look into work recovery-related constructs, such as work recovery experiences and the need for recovery, within the specific context of healthcare professionals (Rus et al., 2020). Considering the high costs of job strain and burnout on the healthcare professionals’ wellbeing and health and on organizational outcomes such as patient safety (Blasche et al., 2017), understanding how to increase wellbeing is imperative.

Considering the new strains that COVID-19 has imposed on the physical, emotional, and cognitive resources of healthcare professionals, we propose that the opportunities afforded to them to take part in work recovery experiences are limited, and hence the resource replenishment process after work is also impaired, resulting in an increased need for recovery and impaired emotional and psychological wellbeing.

Building on previous empirical research (Mohd Fauzi et al., 2020; Alexiou et al., 2021) and theoretical studies (Sonnentag et al., 2022), we consider that healthcare professionals compared to employees from other occupations will report low involvement in work recovery experiences such as psychological detachment, relaxation, mastery, and control and a high level of need for recovery after work. Moreover, the low involvement in the four recovery experiences will result in a high need for recovery after work (Bennett et al., 2017; Steed et al., 2021). Empirical works reveal that the doctors’ high need for recovery leads to intense self-reported health outcomes and poor workplace wellbeing indicated by low life satisfaction, high psychological stress, and low career success (Sun et al., 2021). We thus expect that in the context of the COVID-19 pandemic, the healthcare workers’ wellbeing will suffer. In line with previous calls for investigating specific relationships between work recovery variables and different dimensions of wellbeing (Rus et al., 2020; Steed et al., 2021), we adopted Keyes’s (2002) distinction between emotional and psychological wellbeing to investigate the impact that working in healthcare has on both through work recovery experiences and need for recovery.

Previous findings show that when healthcare professionals are involved in off-work recovery experiences such as psychological detachment, relaxation, mastery, and control, their emotional and psychological wellbeing will be enhanced. For instance, Singh et al. (2016) found that the use of recovery experiences was associated with less exhaustion and psychosomatic symptoms as indicators of psychological wellbeing. Steed et al.’s (2021) meta-analysis revealed that all four recovery experiences were positively related to employees’ mental wellbeing (e.g., low anxiety), the experiences of state affect, life satisfaction, and psychosomatic wellbeing. We expect a similar impact of work recovery experiences on psychological wellbeing as an individual’s optimal functioning in life. Based on the previous research (Wentz et al., 2020), a reduced need for recovery derived from involvement in these off-work recovery experiences will contribute to a high emotional and psychological wellbeing. Thus, we hypothesized as follows:

H1: Recovery experiences of psychological detachment, relaxation, mastery, and control and need for recovery jointly mediate the relationship between working in healthcare and emotional wellbeing.
H2: Recovery experiences of psychological detachment, relaxation, mastery, and control and need for recovery jointly mediate the relationship between working in healthcare and psychological wellbeing.

## Materials and Methods

### Participants

This study was part of the larger COVID-19 IMPACT project^1^, which is an international online survey conducted in 78 countries/regions worldwide exploring the behavioral and psychological impacts of COVID-19 (Dias Neto et al., 2021).

We reported on the data of 217 participants from the Romanian dataset. In our sample, 44 participants (20.28%) were health professionals, while the rest of 173 (79.72%) were employed in other domains. No data on the healthcare professionals’ status in terms of directly working with patients with COVID-19 were collected. Participants’ age ranged between 20 and 63 years (*M* = 31.69, SD = 8.18). Most of them were females (133 participants, 61.29%), and, in one instance, they did not report the gender (46%). Almost half of the participants reported that they were undergraduates (45.16%), and 77 participants (35.48%) have a master’s and/or Ph.D. degree. Less than 20% of the participants reported that they only had a high school diploma or were enrolled in an undergraduate program. The inclusion criterion was the age of at least 18 years. The participants voluntarily participated in this study.

### Measures

The status of working in healthcare was measured with the following item: Do you work in healthcare (as a physician or nurse)? (0 = no vs. 1 = yes).

The Recovery Experience Questionnaire (Sonnentag and Fritz, 2007) measured the following after work recovery experiences: psychological detachment (e.g., “I did not think about work at all”; α = 0.93), relaxation (e.g., “During time after work, I kick back and relax”; α = 0.90), mastery (e.g., “I learned new things”; α = 0.93), and control (e.g., “I feel like I can decide for myself what to do”; α = 0.94). Each recovery experience was measured using four items. Participants indicated their level of agreement on what they do during leisure time using a five-point Likert scale (1 = I do not agree at all, 5 = I fully agree).

Need for recovery after working time was measured with ten items from van Veldhoven and Broersen’s (2003) scale. In line with previous studies (e.g., Sonnentag and Fritz, 2007), the original measurement scale of the items (yes/no) was modified into the following Likert scale: 1 = never, 2 = sometimes, 3 = often, and 4 = always. Participants indicated the extent to which they felt the aspect presented in each statement at the end of the workday during the COVID-19 pandemic (“After the evening meal, I generally feel in good shape”; α = 0.89).

Emotional wellbeing was measured with three items (α = 0.86) from the Mental Health Continuum Short Form (Keyes, 2009). In addition, six items from this instrument measured psychological wellbeing in terms of self-acceptance, environmental mastery, positive relations with others, personal growth, autonomy, and purpose in life (α = 0.88). Each dimension of psychological wellbeing was measured with one item. Participants rated the frequency of every feeling in the past month on a 6-point Likert scale (0 = never, 1 = one time or two times a month, 2 = about one time a week, 3 = two or three times a week, 4 = almost every day, and 5 = every day).

The mean score was considered for each of the scales used. A high scale score indicates a high level of the construct measured.

### Procedure

All the participants provided informed consent before completing the online survey in Google Form. Data were collected from April 15 to May 15, 2021, during the nationwide lockdown. The online survey was distributed by the research team to organizations, students, and academic staff through emails and on social networking websites (e.g., Facebook).

## Results

### Data Analysis

**FIGURE 1:**
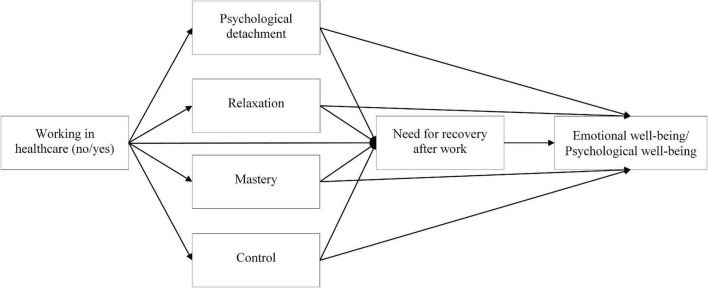
Working model.

To test our hypotheses, two serial mediation analyses were conducted using IBM SPSS Macro PROCESS version 3.5 (Hayes, 2018). We tested a customized model (Figure 1), starting from model 80 (the direct effect of X on Y was set to 0), with the bootstrap technique on 5,000 samples at 95% CI. As the outcome variable, the first mediation analysis considered emotional wellbeing, and the second one, psychological wellbeing. In both analyses, age was included as a covariate as research has shown that it is related to work recovery experiences (Virtanen et al., 2019) and wellbeing (Lawrie et al., 2019).

### Descriptive Analysis

Descriptive statistics (means and SDs), *r* Bravais-Pearson’s correlations, and McDonald’s (ML) ω reliability coefficients are included in Table 1. All the correlations between the variables were consistent with the theorized pattern of relationships, with the exception of the relationships between working in healthcare, on the one hand, and psychological detachment (*r* = −0.12, *p* > 0.05) and need for recovery after work (*r* = 0.09, *p* > 0.05), on the other hand. All the scales had the McDonald’s (ML) ω reliability coefficient higher than 0.70.

**TABLE 1:** Descriptive statistics (means and SDs), *r* Bravais-Pearson’s correlations, and McDonald’s ω reliability coefficients (*N* = 217).

Variable	M	SD	1	2	3	4	5	6	7	8
1. Working in healthcare (no/yes)	–	–	–							
2. Psychological detachment	3.20	1.22	−0.12†	(0.93)						
3. Relaxation	3.67	1.03	−0.15*	0.71***	(0.91)					
4. Mastery	3.59	1.07	−0.14*	0.33***	0.52***	(0.93)				
5. Control	3.87	1.03	−0.14*	0.48***	0.70***	0.63***	(0.94)			
6. Need for recovery	2.06	0.62	0.09	−0.25***	−0.39***	−0.42***	−0.43***	(0.89)		
7. Emotional wellbeing	3.44	1.06	−0.01	0.22***	0.27***	0.36***	0.26***	−0.44***	(0.87)	
8. Psychological wellbeing	3.15	1.12	0.03	0.22***	0.33***	0.45***	0.36***	−0.37***	0.69***	(0.88)

†p < 0.10; *p < 0.05; ***p < 0.001.

### Hypothesis Testing

In the two tested models, the coefficients depicting the paths from working in healthcare (no/yes) to work recovery experiences and need for recovery and from recovery experiences to need for recovery were identical. In this sense, results of the mediation analyses revealed that participants working in healthcare reported lower levels of relaxation (β = −0.41, 95%CI [−0.76; −0.05]), mastery (β = −0.44, 95%CI [−0.79; −0.07]), and control (β = −0.39, 95%CI [−0.75; −0.03]) compared to employees in other domains (Table 2). Working in healthcare was not significantly associated with low psychological detachment (β = −0.31, 95%CI [−0.70; 0.09]) and need for recovery (β = 0.06, 95%CI [−0.14; 0.26]).

**TABLE 2:** Results of the main effect analysis (*N* = 217).

Path	β	SE	LLCI	ULCI
Common in Model 1 (Emotional wellbeing as outcome) and Model 2 (Psychological wellbeing as outcome)				
Working in healthcare (no/yes) → Psychological detachment	−0.31	0.20	−0.70	0.09
Working in healthcare (no/yes) → Relaxation	−0.41	0.18	−0.76	−0.05
Working in healthcare (no/yes) → Mastery	−0.44	0.19	−0.79	−0.07
Working in healthcare (no/yes) → Control	−0.39	0.19	−0.75	−0.03
Working in healthcare (no/yes) → Need for recovery	0.06	0.10	−0.14	0.26
Psychological detachment → Need for recovery	0.01	0.04	−0.09	0.10
Relaxation → Need for recovery	−0.09	0.07	−0.22	0.03
Mastery → Need for recovery	−0.13	0.05	−0.23	−0.03
Control → Need for recovery	−0.11	0.06	−0.22	0.00
**Model 1: Emotional wellbeing as outcome**				
Psychological detachment	0.11	0.08	−0.05	0.27
Relaxation	−0.02	0.12	−0.25	0.21
Mastery	0.21	0.08	0.07	0.37
Control	−0.06	0.09	−0.24	0.11
Need for recovery	−0.58	0.12	−0.81	−0.33
**Model 2: Psychological wellbeing as outcome**				
Psychological detachment	0.05	0.08	−0.11	0.20
Relaxation	0.03	0.13	−0.25	0.28
Mastery	0.31	0.08	0.16	0.47
Control	0.07	0.11	−0.13	0.28
Need for recovery	−0.30	0.13	−0.55	−0.06

Psychological detachment, relaxation, and control were not significantly associated with the need for recovery (β = 0.01, 95%CI [−0.09; 0.10]; β = −0.09, 95%CI [−0.22; 0.03]; β = −0.11, 95%CI [−0.22; 0.00]). Only a high level of mastery was related to a low need for recovery after work (β = −0.13, 95%CI [−0.22; 0.00]).

In addition, psychological detachment, relaxation, and control were not significantly related to emotional wellbeing (β = 0.11, 95%CI [−0.05; 0.27]; β = −0.02, 95%CI [−0.25; 0.21]; β = −0.06, 95%CI [−0.24; 0.11]). Instead, mastery was positively related to emotional wellbeing (β = 0.21, 95%CI [0.07; 0.37]). A high need of recovery after work was related to a low emotional wellbeing (β = −0.58, 95%CI [−0.81; −0.33]).

Similar results were obtained regarding the relationships between work recovery experiences, need for recovery after work, and psychological wellbeing. Specifically, psychological detachment, relaxation, and control were not significantly related to psychological wellbeing (β = 0.05, 95%CI [−0.11; 0.20]; β = 0.03, 95%CI [−0.25; 0.28]; β = 0.07, 95%CI [−0.13; 0.28]). High mastery was related to a high psychological wellbeing (β = 0.31, 95%CI [0.16; 0.47]). Also, need for recovery was negatively correlated with psychological wellbeing (β = −0.30, 95%CI [−0.55; −0.06]).

#### Hypothesis 1

Although the total indirect effect of working in healthcare on emotional wellbeing was significant (β = −0.20, 95%CI [−0.38; −0.04]), at the individual level, only two paths were significant (Table 3). Among the recovery experiences considered, only mastery significantly intervenes between working in healthcare and emotional wellbeing (β = −0.09, 95%CI [−0.21; −0.01]). The other recovery from work experiences do not mediate the relationship between working in healthcare and emotional wellbeing (psychological detachment, β = −0.03, 95%CI [−0.12; 0.02]; relaxation, β = 0.01, 95%CI [−0.10; 0.13]; control, β = 0.02, 95%CI [−0.05; 0.12]). In addition, the need for recovery did not act as a mediator between working in healthcare and emotional wellbeing (β = −0.04, 95%CI [−0.16; 0.08]). Only mastery and need for recovery jointly mediated the relationship between working in healthcare and emotional wellbeing (β = −0.03, 95%CI [−0.08; −0.002]). The other paths were not statistically significant. Thus, our first hypothesis received partial empirical support.

**TABLE 3:** Total and individual indirect effects of working in healthcare on emotional and psychological wellbeing through work recovery experiences and need for recovery (*N* = 217).

	Emotional wellbeing				Psychological wellbeing			
Path	b	SE	LLCI	ULCI	b	SE	LLCI	ULCI
Total indirect effect	−0.20	0.09	−0.38	−0.04	−0.25	0.09	−0.44	−0.07
Working in healthcare (no/yes) → Psychological detachment	−0.03	0.04	−0.12	0.02	−0.02	0.03	−0.09	0.04
Working in healthcare (no/yes) → Relaxation	0.01	0.05	−0.10	0.13	−0.01	0.06	−0.13	0.12
Working in healthcare (no/yes) → Mastery	−0.09	0.05	−0.21	−0.01	−0.14	0.07	−0.30	−0.02
Working in healthcare (no/yes) → Control	0.02	0.04	−0.05	0.12	−0.03	0.05	−0.13	0.06
Working in healthcare (no/yes) → Need for recovery	−0.04	0.06	−0.16	0.08	−0.02	0.04	−0.09	0.05
Working in healthcare (no/yes) → Psychological detachment → Need for recovery	0.00	0.01	−0.02	0.02	0.00	0.01	−0.01	0.01
Working in healthcare (no/yes) → Relaxation → Need for recovery	−0.02	0.02	−0.07	0.01	−0.01	0.01	−0.04	0.01
Working in healthcare (no/yes) → Mastery → Need for recovery	−0.03	0.02	−0.08	−0.002	−0.02	0.01	−0.05	−0.0002
Working in healthcare (no/yes) → Control → Need for recovery	−0.03	0.02	−0.07	0.003	−0.01	0.01	−0.05	0.00

#### Hypothesis 2

Regarding the effect of working in healthcare on psychological wellbeing, results indicate a significant total indirect effect (β = −0.25, 95%CI [−0.44; −0.07]; Table 3). Only two paths were statistically significant. Specifically, mastery was a mediator in the relationship between working in healthcare and psychological wellbeing (β = −0.14, 95%CI [−0.30; −0.02]). Psychological detachment, relaxation, control, and need for recovery did not significantly intervene between working in healthcare and psychological wellbeing (β = −0.02, 95%CI [−0.09; 0.04]; β = 0.01, 95%CI [−0.13; 0.12]; β = 0.03, 95%CI [−0.13; 0.06]; β = 0.02, 95%CI [−0.09; 0.05]). Also, jointly with the need for recovery, they did not mediate the relationship between working in healthcare and psychological wellbeing. Only mastery and need for recovery jointly intervened in the relationship between working in healthcare and psychological wellbeing (β = −0.02, 95%CI [−0.05; −0.0002]). Thus, the second hypothesis was partially empirically supported.

## Discussion

We explored how the four recovery experiences (i.e., psychological detachment, relaxation, mastery, and control) as antecedents of the need for recovery act as mediators in the relationship between working in healthcare and different dimensions of wellbeing (i.e., emotional and psychological wellbeing). The research model was found to have a full mediation effect both in the case of emotional and psychological wellbeing. However, this effect was carried out mainly by mastery and mastery as an antecedent of the need for recovery. Mastery experiences as off-job activities that distract from the job by providing challenging experiences and learning opportunities in other domains. It seems only they can reduce participants’ desire for being temporarily relieved from demands in order to recuperate and to replenish their internal resources. By offering opportunities for experiencing competence and proficiency, mastery experiences generate directly, and indirectly through need for recovery high happiness, positive emotions, satisfaction with life in general (i.e., emotional wellbeing), and optimal functioning in private life (i.e., psychological wellbeing).

These findings highlight the crucial roles of mastery as recovery from work experience and the need for recovery in linking working in healthcare with emotional and psychological wellbeing. At present, no empirical research integrated the off-work recovery experiences, need for recovery, and different dimensions of wellbeing in healthcare professionals, despite the recent calls in the literature on this topic (Rus et al., 2020; Steed et al., 2021). Compared to previous research, by focusing on the emotional and psychological dimensions of wellbeing, we also considered the positive side of healthcare professionals’ wellbeing during the COVID-19 pandemic (Lai et al., 2020; Liu et al., 2020; Rossi et al., 2020; Caldas et al., 2021; Wang et al., 2021).

Our data shows that psychological detachment, relaxation, and control as work recovery experiences were not the significant predictors of the two dimensions of global wellbeing. It is possible that these relationships are mediated by domain wellbeing (Newman et al., 2014), such as workplace-related psychological wellbeing (Parker and Hyett, 2011).

The fact that healthcare professionals in our sample reported lower levels of recovery experiences compared to other professionals is in line with other recent studies that showed that medical personnel enjoys limited recovery experiences to recover from their job demands during the COVID-19 pandemic (Mohd Fauzi et al., 2020). As healthcare professionals did not report higher levels of need for recovery than other professionals, we expect that may be due to intervening variables such as occupational calling. Recent research shows that occupational calling is a critical psychological driving force that keeps healthcare professionals (i.e., nurses) focused and motivated despite tremendous challenges (Zhu et al., 2021).

Our study contributes to the literature on work recovery experiences by answering the call from Rus et al. (2020) to investigate the antecedents of work recovery experiences specifically in healthcare professionals. By examining the need for recovery and different dimensions of wellbeing as potential outcomes of work recovery experiences, we added empirical evidence to the small body of research investigating the benefits and pitfalls of work recovery experiences in healthcare professionals compared to other occupations. In addition, we offered empirical evidence that the four work recovery experiences, although they are positively associated, are empirically distinctive. By focusing on the need for recovery as a specific aspect of impaired wellbeing (Sonnentag and Fritz, 2007) and the emotional and psychological wellbeing as the dimensions of positive mental health (Keyes, 2002), this study provides an integrative understanding of the healthcare professionals’ wellbeing. In addition, it extends the current knowledge on different mediating paths through which the status of working in healthcare relates to emotional and psychological wellbeing.

This study provides additional information about the contexts where recovery experiences need for recovery and wellbeing research takes place. Although healthcare contexts provide an area of high interest when studying these concepts separately, little of the published research integrate them with a sample of professionals, in general, and even less with healthcare professionals (Dai et al., 2020), particularly from Eastern Europe.

Our findings have several implications for practice. Top management should consider improving work in healthcare as our findings revealed that healthcare professionals report lower levels of relaxation, mastery, and control after work compared to employees from other work domains. As Bennett et al. (2017) meta-analysis showed both work characteristics and after-work recovery play an important role in determining employee wellbeing, mainly emotional wellbeing. Healthcare professionals should be encouraged to experience mastery in order to reduce their need for recovery and to increase both emotional and psychological wellbeing. They should engage in activities such as social, creative, physical, and low-effort activities that facilitate recovery experiences (de Jonge et al., 2018), especially mastery (Tuisku et al., 2016). As shown, recovery experiences including mastery can be increased through training (Siu et al., 2014).

Our study has several limitations. First, there was an imbalance between the percentage of the healthcare professionals included in the sample (20.28%) and that of the employees working in other fields (78.72%). Second, the healthcare professionals participating in our study derived from more than one organization. We did not collect data on the type of organizations from which the participants were derived. During the nationwide lockdown, only some hospitals and clinics were approved to take care of the patients infected with severe acute respiratory syndrome coronavirus 2 (SARS-CoV-2). It is possible that not all healthcare professionals dealt with the challenges imposed by the COVID-19 outbreak at their workplaces. Future studies should consider larger representative samples.

Our self-reported cross-sectional data prevent us from inferring causal relationships. Building on previous research (Janicke et al., 2017), it would be interesting to use research designs that adopt a within-subject approach and permit consideration of feedback loops or bidirectional causal influences between the variables.

We did not include the social dimension of wellbeing from Keyes’s (2002, 2009) model. Furthermore, we considered the global scores of psychological wellbeing although Keyes’s (2009) model reveals that both social and psychological wellbeing has multiple dimensions. It is possible that different recovery from work experiences and the need for recovery tap differently into distinct dimensions of social and psychological wellbeing. In addition, other recovery experiences such as meaning and affiliation (Newman et al., 2014) can be studied in relation to different dimensions of wellbeing.

## Data Availability Statement

The raw data supporting the conclusions of this article will be made available by the authors, without undue reservation.

## Ethics Statement

Ethical review and approval was not required for the study on human participants in accordance with the Local Legislation and Institutional Requirements. The patients/participants provided their written informed consent to participate in this study.

## Author Contributions

CR, CV, CO, AK, and MK: designing study. CR, CO, and AG: literature review and data analysis. CR, CO, AB, CV, AK, and MK: discussing the results. CR and CO: writing the manuscript. All authors contributed to the article and approved the submitted version.

## Conflict of Interest

The authors declare that the research was conducted in the absence of any commercial or financial relationships that could be construed as a potential conflict of interest.

## Publisher’s Note

All claims expressed in this article are solely those of the authors and do not necessarily represent those of their affiliated organizations, or those of the publisher, the editors and the reviewers. Any product that may be evaluated in this article, or claim that may be made by its manufacturer, is not guaranteed or endorsed by the publisher.

## Footnotes

1
https://ucy.ac.cy/acthealthy/en/covid-19-impact-survey
